# Fabrication, Optimization and Characterization of Natural Dye Sensitized Solar Cell

**DOI:** 10.1038/srep41470

**Published:** 2017-01-27

**Authors:** William Ghann, Hyeonggon Kang, Tajbik Sheikh, Sunil Yadav, Tulio Chavez-Gil, Fred Nesbitt, Jamal Uddin

**Affiliations:** 1 Center for Nanotechnology, Department of Natural Sciences, Coppin State University, 2500 W. North Ave, Baltimore, MD, USA

## Abstract

The dyes extracted from pomegranate and berry fruits were successfully used in the fabrication of natural dye sensitized solar cells (NDSSC). The morphology, porosity, surface roughness, thickness, absorption and emission characteristics of the pomegranate dye sensitized photo-anode were studied using various analytical techniques including FESEM, EDS, TEM, AFM, FTIR, Raman, Fluorescence and Absorption Spectroscopy. Pomegranate dye extract has been shown to contain anthocyanin which is an excellent light harvesting pigment needed for the generation of charge carriers for the production of electricity. The solar cell’s photovoltic performance in terms of efficiency, voltage, and current was tested with a standard illumination of air-mass 1.5 global (AM 1.5 G) having an irradiance of 100 mW/cm^2^. After optimization of the photo-anode and counter electrode, a photoelectric conversion efficiency (*η*) of 2%, an open-circuit voltage (*Voc*) of 0.39 mV, and a short-circuit current density (*Isc*) of 12.2 mA/cm^2^ were obtained. Impedance determination showed a relatively low charge-transfer resistance (17.44 Ω) and a long lifetime, signifying a reduction in recombination losses. The relatively enhanced efficiency is attributable in part to the use of a highly concentrated pomegranate dye, graphite counter electrode and TiCl_4_ treatment of the photo-anode.

Solar energy is rapidly becoming the most viable, eco-friendly and sustainable alternate source of renewable energy^1^. There has been an exponential growth in the production of solar cells over the past two decades^1^,^2^,^3^. One of the most promising means of solar energy conversion is the use of dye-sensitized solar cells (DSSC) first developed by Grätzel *et al*. in 1991 and has attracted much attention in the last couple of decades leading to a plethora of publications on the subject^4^,^5^,^6^,^7^,^8^,^9^,^10^. The Interest in DSSC and more precisely Natural Dye Sensitized Solar Cell (NDSSC) is mainly due to its low cost of fabrication and environmental friendly nature^11^,^12^.

Currently, DSSC has the potential to convert photons from sunlight to electrical energy at 13% efficiency^13^. An intensive effort has been directed towards the optimization of the various components of DSSC with the goal of fabricating more efficient and stable cells. The DSSC as displayed in Fig. 1 is composed of a redox couple electrolyte system, typically iodide and triiodide complex, sandwiched between two glass plates, a photo-anode and a counter electrode. The redox couple in the electrolyte is used to regenerate the oxidized dye. The photo-anode consist of glass plate coated with a thin layer of transparent conductive oxide (Fluorine doped tin-oxide, FTO or Indium doped tin-oxide, ITO) and a monocrystalline semiconductor (usually titanium dioxide, TiO_2_) which serves as a scaffold for the dye sensitizer. The counter electrode is also made up FTO covered glass slide coated usually with a thin film of Platinum or Carbon for the catalysis of the redox reaction with electrolyte.

The total efficiency of the DSSC is dependent on the nature, optimization and compatibility of each one of the components of the solar cell and more particularly the photo-anode which plays a vital role in the charge generation and transfer processes. To increase the power conversion efficiency of DSSC, a nanostructured porous titanium dioxide with a wide band gap and large exciton binding energy is typically used in constructing the photo-anode. The large surface area of the nanostructured TiO_2_ insures the adsorption of sufficiently large number number of dye molecules for efficient harvesting of radiant energy. The dye, typically, with a wide and intense absorption spectrum is adsorbed on the TiO_2_ via anchoring groups such as carboxylic, carbonyl and hydroxyl located on the dye molecule. A strong adsorption of the dye unto the nanostructured TiO_2_ is required for the efficient electron injection into the conduction band of the TiO_2_ semiconductor.

The dye sensitizer anchored on the surface of titanium dioxide absorbs photons and gets electronically excited. Energy is produced when the excited electron moves from the valence band to the conduction band of the semiconductor. The electrolyte (I^−^/I_3_^−^) donates electron back to the dye. The sensitizers currently used in the fabrication of solar cells are transition metal coordination complexes such as Ruthenium (II) carboxylated polypyridyl complexes, due to their intense charge-transfer absorption in the whole visible range and highly efficient metal-ligand charge transfer transition (MLCT)^14^,^15^. Natural dyes are however more desirable than these synthetic dyes because they are more economical, easily attainable, and abundant in supply and environmentally friendly^5^,^16^. In addition, they turn to have large absorption coefficient owing to allowed π to π* transitions.

The performance of various natural dye containing pigments such as anthocyanin^17^,^18^, xanthene^14^, chlorophyll, carotenoid and flavonoid have been thoroughly investigated^11^. These pigments are derived from various plant parts such as flower petals, leaves, roots and fruits pulp/bark.

In this study, juice extracted from pomegranate was investigated as potent sensitizer for DSSC in comparison to other natural dyes. The pomegranate is a rich source of anthocyanin and we have deployed it as a sensitizer for the absorption of photons in DSSCs in the generation of electricity^19^. Anthocyanins are polyphenolic ring-based flavonoids widely known for their antioxidant properties^20^. They differ from other flavonoids by the presence of a positive charge in the central ring structure and are responsible for the intense colors of other fruits and vegetables such as the berries and red cabbages^20^. They have been shown to display a wide absorption/emission band in the UV-Visible region of the spectrum as a result of electron charge transfer transitions^16^,^21^.

## Results and Discussion

### Absorption spectra

**Figure 2:**
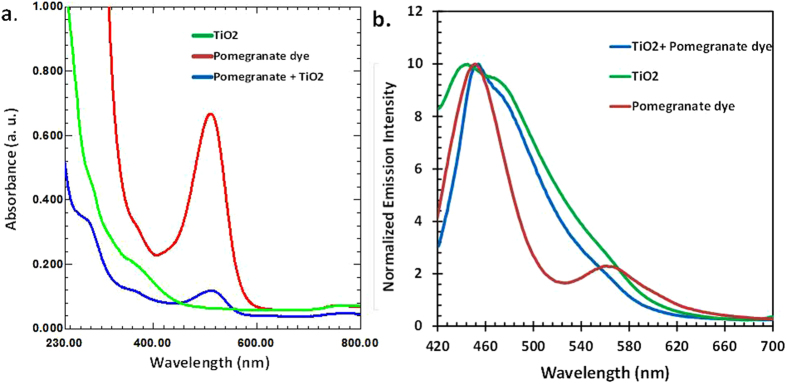
Absorption and emission spectra of bare titanium dioxide (green), pomegranate dye extract (red) and pomegranate sensitized TiO_2_ film on FTO glass (blue). (**a**) Absorption spectra; (**b**) emission spectra.

The overall performance of a DSSC is reliant on the light absorption capability of the dye sensitizer and the diffusion of the ejected electron through the mesoporous TiO_2_ film. The optical properties of the dye extracted from pomegranate were investigated using UV-vis spectrometry. The absorption spectra of pomegranate dye extract, bare TiO_2_ and pomegranate dye/TiO_2_ films are illustrated in Fig. 2a. The Pomegranate dye extract exhibited an intense absorption broad band in the visible region with a peak at 510 nm. This intense absorption in the visible region (510 nm) matches with that reported for anthocyanin and is the reason for the efficient harvesting of photons in NDSSC^19^. Plant parts containing anthocyanins have been widely investigated for use as photosensitizers in the fabrication of NDSSC. In addition to their high absorption coefficient in the visible region of the electromagnetic spectrum, the presence of hydroxyl and carbonyl anchoring groups on anthocyanins enable their adsorption unto the surface of the TiO_2_. This adsorption facilitates the transfer of the injected electron from dye to the conduction band of TiO_2_ which ultimately enhances the efficiency of NDSSC^5^. A non-zero absorbance was observed for the spectrum corresponding to pomegranate dye/TiO_2_. The peak for anthocyanin in Pomegranate dye/TiO_2_ is not easily visible in the spectrum, nonetheless, a shift in the maximum wavelength is observed. The absorption band of the pomegranate dye has been reported to be red shifted to about 550 nm upon chelation to TiO_2_^22^. The attachment of the anthocyanin containing dye onto TiO_2_ has been suggested to have an effect on the HOMO and LUMO level of anthocyanin, which consequently decreases the band gap resulting in a redshift of the absorption peak^23^. The peak in the ultra-violet region is prominent and shows a bathochromic shift from 275 nm to 285 nm. A non-zero absorption similar to that of pomegranate dye/TiO_2_ was also observed in spectrum of bare TiO_2_. The non-zero absorption has been attributed to diffused reflection of TiO_2_ suspended in solvent. Due to the wideband gap of TiO_2_ (3.2 eV), bare TiO_2_ do not absorb visible light, hence, no absorption band is seen in the region extending from 400 nm to 700 nm as exhibited in Fig. 2a. Figure 2b shows the fluorescence spectra of pomegranate dye, pomegranate dye/TiO_2_ and bare TiO_2_ employed in the fabrication of the NDSSC. The measurement was carried out with the exciting light in the range of 320 nm ≤ λ_exc_ ≤ 600 nm.

### Fourier Transformed Infrared (FTIR) Analysis

**Figure 3:**
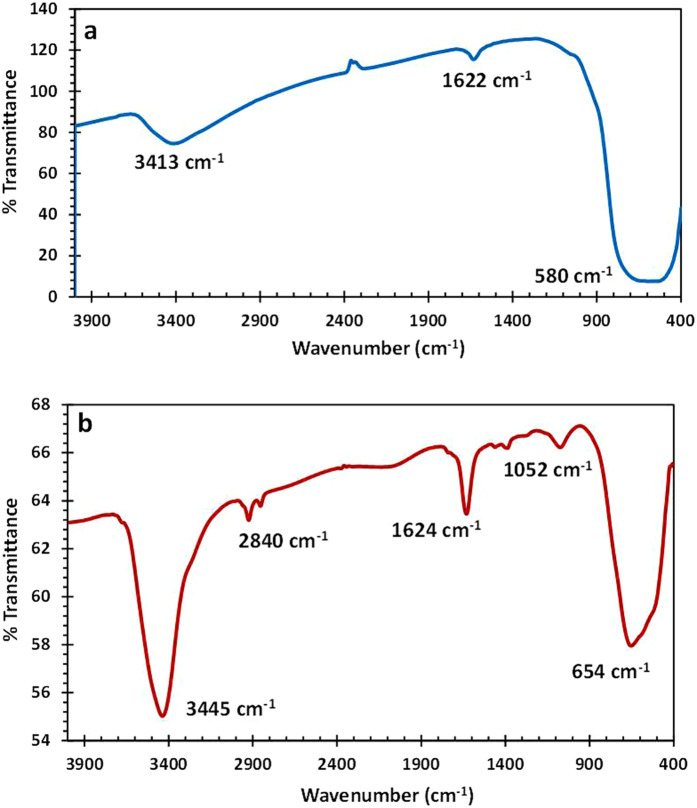
FT-IR spectrum of (**a**) TiO_2_ and (**b**) TiO_2_-POM film in KBr pellet.

The IR spectra of the films annealed at room temperature shows three vibrational modes (Fig. 3a) which are ascribed to TiO_2_ for υ_O-H_ at 3413 cm^−1^, δ_O-H_ at 1622 cm^−1^, and υ_Ti-O_ at 580 cm^−1^ as a broad stretching. The presence of pomegranate organic groups (Fig. 3b) such as hydroxyl, ethyl, acetyl and ethoxide in the films^23^, shows stretching’s for υ_OH_ at 3649 cm^−1^, υ_O-H(water)_ at 3445 cm^−1^, υ_CH2_, υ_CH3_ at 2912, 2840 cm^−1^, δ_CH2_, δ_CH3_ at 1463, 1367 cm^−1^, δ_C-C_ at 1710 cm^−1^, δ_C=Cbenz_ at 1627 cm^−1^, υ_C–O_ at 1052 cm^−1^, and υ_Ti-O-C_ at 654 cm^−1^, respectively. An interesting observation in these studies was that the stretching intensities were increased with the deposition of pomegranate dye, with a significant change on the TiO_2_ IR modes, which indicates that there occurred an interaction that shift to low energies the initially TiO_2_ stretching. This observation shows that the deposition of pomegranate dye undergoes strong phase transition due to an electronic environment change around the TiO_2_ energies^24^,^25^,^26^.

### Raman Spectroscopy

**Figure 4:**
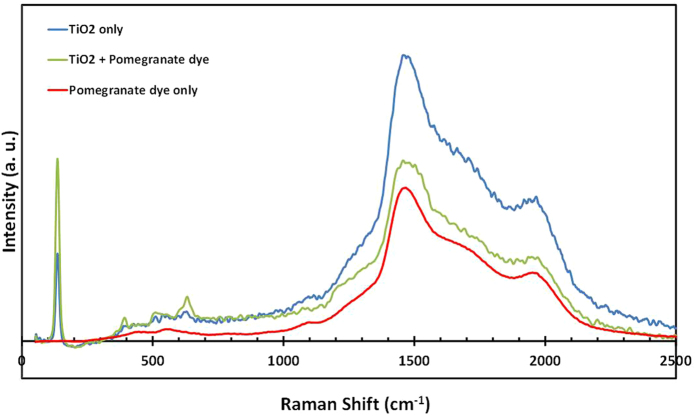
Raman spectra of sample films at room temperature of: TiO_2_ (blue); and TiO_2_ + Pomegranate dye (green); Pomegranate dye (red).

To further evaluate the Pomegranate dye/TiO_2_ interaction in the nanocrystalline prepared films, Raman studies were performed in the range of 0–2500 cm^−1^ and the results are shown in Fig. 4. The D and G bands at 1470 cm^−1^ and 1960 cm^−1^, respectively, have been previously attributed to the high disorder encountered for sp^3^ carbons^27^,^28^. The intense peak at 130 cm^−1^ assigned E_(g)_ is present in both of TiO_2_ (blue) and the hybrid of TiO_2_ and pomegranate (green), but it is absent in the measurement for pomegranate dye only. However, an interesting observation is that this 130 cm^−1^ peak, which is as a result of the symmetric stretching vibration of O−Ti−O in TiO_2_, is enhanced after the adsorption of the pomegranate dye. This observation proves that there is an interaction between the TiO_2_ nanoparticles and the pomegranate dye. We speculate that the adsorbed pomegranate dye excited by the light source causes the enhancement of the E_(g)_ peak of TiO_2_.

### Morphological Studies of the Fabricated Pomegranate Sensitized Solar Cell

**Figure 5:**
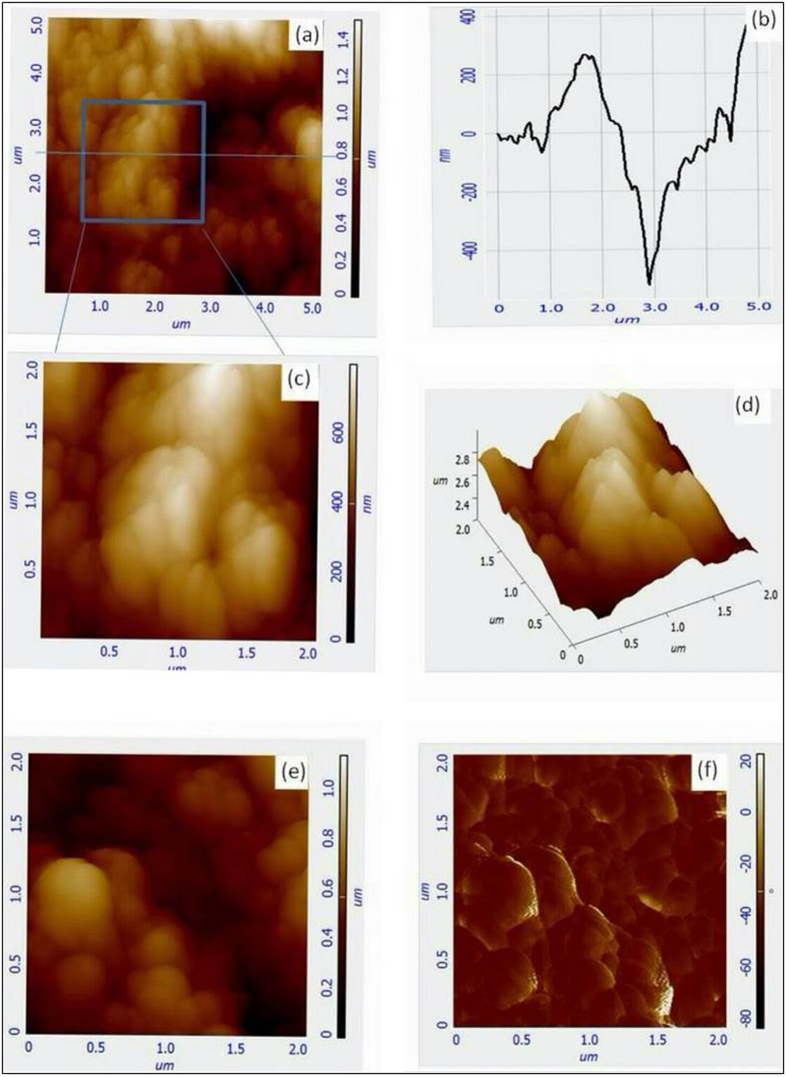
The AFM images of the bare TiO_2_ and Dye/TiO_2_ samples. (**a**) Topographic image of TiO_2_ (blue line for the cross-section analysis) in 5 μm × 5 μm; (**b**) Image profile of the TiO_2_ topographic image; (**c**,**d**) 2D and 3D Topographic images of TiO_2_ in 2 μm × 2 μm, respectively; (**e**,**f**) 2D Topographic and Phase images of Dye/TiO_2_ in 2 μm × 2 μm, respectively.

Atomic force microscopy (AFM) images of the TiO_2_ electrode, before and after the addition of pomegranate dye extract were obtained in order to examine the surface morphological characteristics of the nanocrystalline TiO_2_. The Fig. 5 shows the AFM images of bare TiO_2_ and pomegranate dye sensitized TiO_2_ on FTO glass (dye/TiO_2_). The images were obtained in 5 μm × 5 μm and 2 μm × 2 μm areas (Fig. 5a and b), analyzed to obtain insight the roughness and topographic features of the samples. Details of the surface structure of TiO_2_ at the nanometer scale were observed. A high degree of roughness was observed on the surface of both bare TiO_2_ and pomegranate dye/TiO_2_. The Root mean square roughness of bare TiO_2_ was about 197.6 nm in the 5 μm × 5 μm scan area (Fig. 5a). Many TiO_2_ clusters and cracks were found as exhibited in the topographic image and the image profile shown in Fig. 5(a–e). The rough surface of the TiO_2_ increases the surface area of the film enabling more pomegranate dye to be adsorbed, which consequently increases the absorption of the incident light. The color distribution in the phase image of the dye/TiO_2_ was very uniform as shown in Fig. 5f, which is an indication that the pomegranate dye is uniformly distributed over the whole TiO_2_ layer.

### Field Emission Scanning Electron Microscopy Imaging

**Figure 6:**
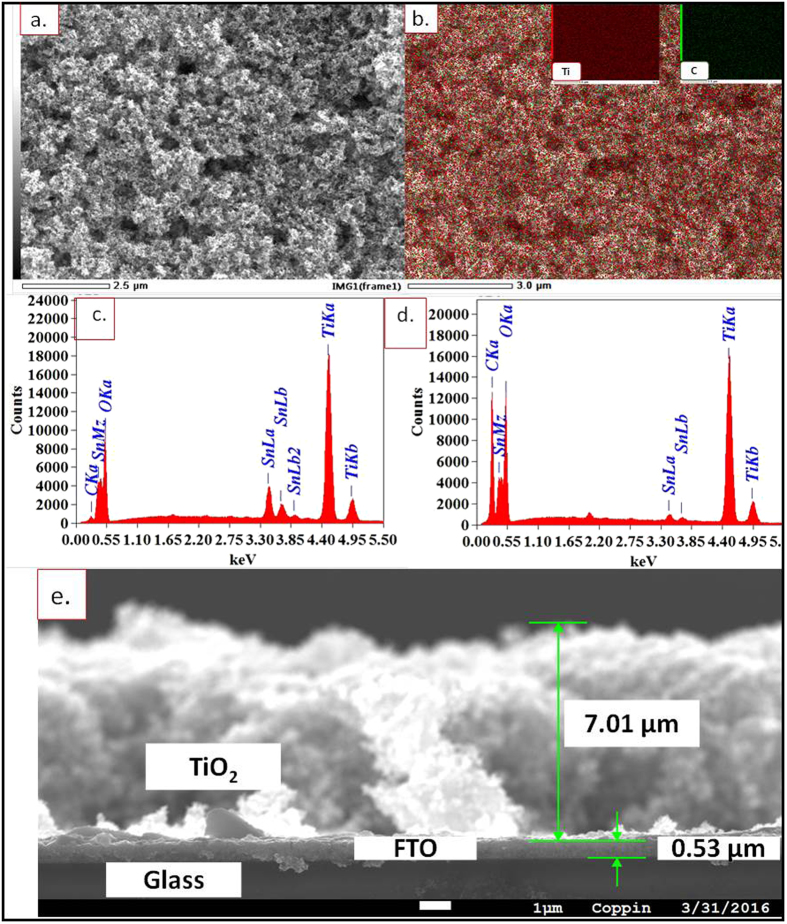
FESEM images of TiO_2_ and Dye/TiO_2_ samples. (**a**) Pomegranate dye sensitized FTO slide; (**b**) map analysis of pomegranate dye sensitized FTO slide; (**c**) EDS of bare TiO_2_ coated FTO slide; (**d**) EDS of pomegranate sensitized FTO slide; (**e**) cross section of TiO_2_ coated FTO slide.

The morphological characteristics and elemental analysis of the TiO_2_ photo-anode were investigated using Field-Emission Scanning Electron Microscopy (JSM 7100F, JEOL.COM) and Energy dispersive X-ray spectroscopy (EDS), respectively. No significant changes were observed in the surface morphology of the samples before and after dye sensitization. The Fig. 6a displays the FESEM image of the TiO_2_ film with an adsorbed layer of pomegranate dye (pomegranate dye/TiO_2_). A highly porous network of nanocrystalline TiO_2_ on FTO glass can be observed from the FESEM image. The EDS mapping analysis of the pomegranate dye/TiO_2_ (Fig. 6b) revealed the presence of carbon and Titanium, corresponding to the major elements present in pomegranate dye and TiO_2_, respectively. The carbon content of pomegranate dye/TiO_2_ in comparison with the bare TiO_2_ was also investigated with EDS. The relatively intense carbon peak in the spectra of the dye-sensitized TiO_2_ film (Fig. 6d) compared to the negligible carbon peak in spectra of the bare TiO_2_ film (Fig. 6c) is indicative of a thorough adsorption of pomegranate dye onto the TiO_2_ film. Although carbon and oxygen are the structural elements of the pigments present in the pomegranate dye, the peaks for oxygen are relatively the same in both EDS spectra (Fig. 6c and d) due to the oxygen component of TiO_2_.

The Fig. 6e shows a cross sectional view of the Dye/TiO_2_ sample exhibiting distinct layers of TiO_2_, FTO and the glass substrate. The elemental composition of the layers was confirmed by the EDS analysis and is displayed in Supplementary Fig. 7. The thickness of FTO on the glass was found to be about 500 nm (nominated size: 400 nm) and Dye/TiO_2_ film was found to be approximately 7 μm.

As compared to the TiO_2_ thin films prepared with other techniques such as the electrophoretic deposition method and the chemical reduction method, the TiO_2_ film prepared by the spin coating method tend to possess a high void content, ample surface irregularities and a high degree of roughness, consistent with the findings of this imaging studies^29^,^30^. Thus, the porous surface of the TiO_2_ photo-anode suggests an enhancement in the adsorption of pomegranate dye into TiO_2_ structure.

### Transmission Electron Microscopy Imaging

**Figure 7:**
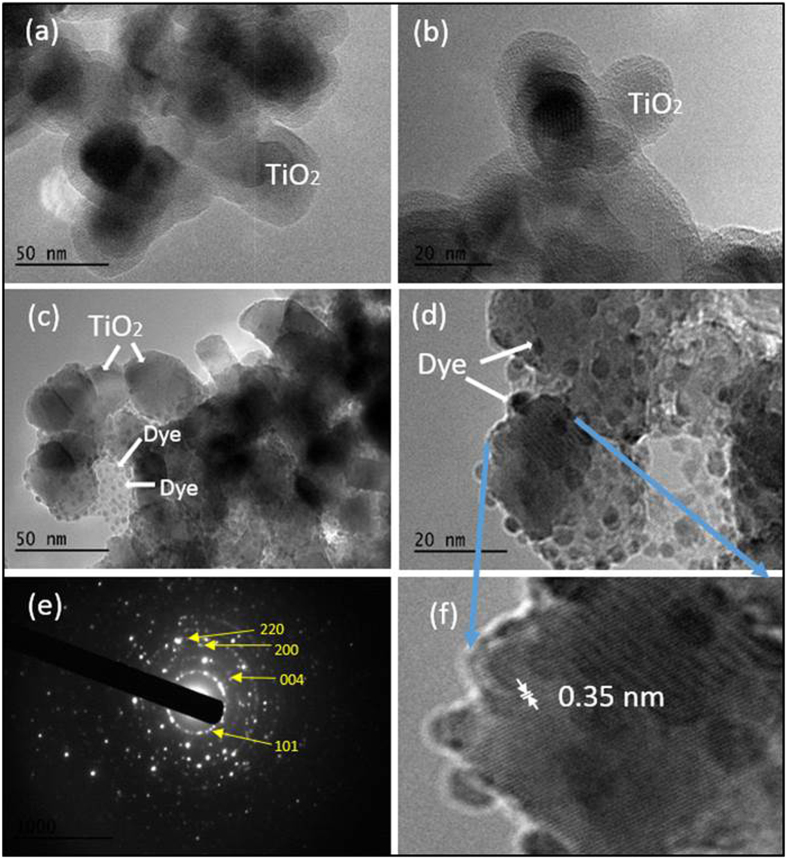
High-resolution TEM images of a TiO_2_ and a pomegranate dye/TiO_2_ samples and a diffraction image of the TiO_2_: (**a**,**b**) high resolution TEM images of TiO_2_, (**c**,**d**) TEM of dye/TiO_2_, (**e**) SAED pattern, (**f**) a zoom-in image showing the d-spacing value of the TiO_2_.

The Transmission electron microscopy (TEM) images of the bare TiO_2_ nanoparticles and the pomegranate dye sensitized TiO_2_ nanoparticles are displayed in Fig. 7. A monodispersed layer of spherical TiO_2_ nanoparticles with particle size in the range of 20–25 nm was observed (Fig. 7a and b). The Fig. 7c and d clearly show the presence small spherical nanoparticles, likely dye particles, with size in the range of 1–3 nm well distributed and decorated on the surface of the TiO_2_ particles with a lattice fringe. These spherical nanoparticles were not observed on the bare TiO_2_ nanoparticles as seen in the Fig. 7a and b which leads us to speculate that they are indeed dye particles. The interaction between the pomegranate dye and the TiO_2_ nanoparticles results from the deprotonation of hydroxyl groups from the anthocyanin pigment.

The selected area electron diffraction (SAED) pattern of the dye/TiO_2_ film as displayed in Fig. 7(e) shows the crystalline nature of the TiO_2_ nanoparticles. The four bright concentric diffraction rings in the SAED can be indexed as the (101), (004), and (200) planes of the anatase TiO_2_, and (220) plane of the rutile TiO_2_. The HRTEM image of the TiO_2_/dye (Fig. 7d), displays the lattice fringe with an interlayer spacing of 0.3487 corresponding to the d-spacing of (101) planes in anatase TiO_2_ as shown in the Fig. 7f.

### Solar conversion efficiency measurement

**Figure 8:**
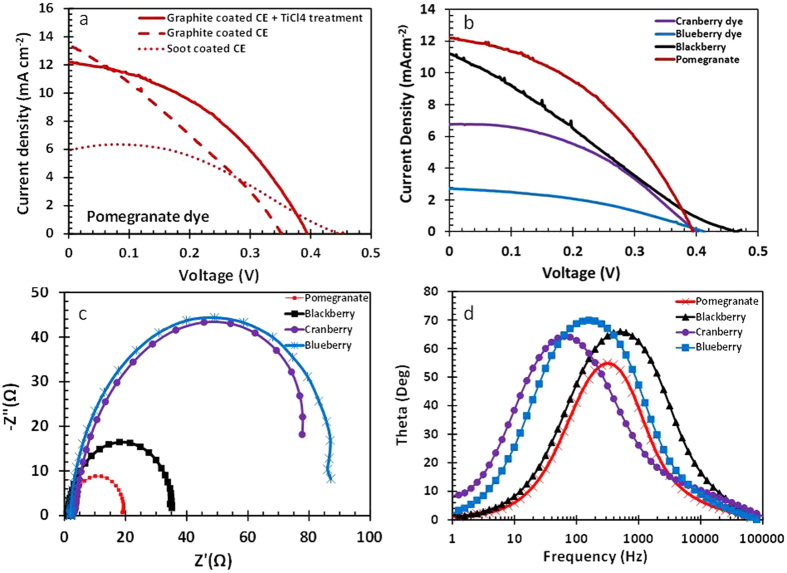
Photocurrent-voltage characteristics for dye sensitized solar cell measured under illumination of 100 W/cm^2^ (1.5 AM): (**a**) Pomegranate dye sensitized solar cell with different electrodes; (**b**) Comparison of Pomegranate dye sensitized solar with other dyes; (**c**) Nyquist of different dye sensitized cells; (**d**) and Bode plots of Pomegranate, Blackberry, Cranberry and Blueberry dye sensitized cells.

The solar cell’s photovoltic performance in terms of efficiency, voltage, and current was tested with a standard illumination of air-mass 1.5 global (AM 1.5G) having an irradiance of 100 mW/cm^2^. After optimization of the photoanode and counter electrode, a photoelectric conversion efficiency (*η*) of 2%, an open-circuit voltage (*Voc*) of 0.39 mV, and a short-circuit current density (*Isc*) of 12.2 mA/cm^2^ were obtained. The enhanced cell efficiency is attributed to the robust dye purification procedures adopted, treatment of anode with TiCl_4_ and the use of graphite counter electrode. A further treatment of TiO_2_ coated FTO glass with TiCl_4_ has been shown to provide more sites for dye adsorption leading to high dye concentration that ensure the absorption of a greater amount of sunlight. The earlier fabrication carried out with just amorphous carbon and colloidal graphite (Fig. 8a) yielded 1.16% and 1.41% efficiencies, respectively. The improved efficiency obtained is also higher than a 1.5% efficiency of a pomegranate sensitized solar cell previously published by Bazargan *et al*.^22^. This efficiency is also higher than most natural dye sensitized solars as revealed in a review by Hug *et al*.^11^ and other reviews in which percent efficiency of solar cell made from natural dyes were mostly less than one.

The performance of the pomegranate dye as a potent sensitizer assessed in comparison with other dyes via the current, voltage, fill factor and conversion efficiency measurement are displayed in Table 1. The data shows that pomegranate sensitizer efficiency is far greater than that of berry fruit dyes employed in the study. The natural dyes studied included Blackberry, Cranberry and Blueberry dyes. Blackberry dye sensitized solar cell had the second highest efficiency (1.4%) followed by Cranberry sensitized solar cell (1.2%) and Blueberry dye sensitized solar cell (0.40%).

### Electrochemical Impedance Analysis

The electrochemical impedance spectroscopy (EIS) has often been used to probe the kinetics and energetics of charge transport and recombination in dye sensitized solar cells. The EIS were recorded in the frequency range between 1 Hz and 100 KHz. Figure 8c shows the Nyquist plot of the various dye sensitized solar cells. Well-defined semicircles related to the charge transfer resistance between the counter electrode and redox (I^−^/I_3_^−^) electrolyte are shown in the high frequency regions. In the EIS analysis, the pomegranate dye solar cell shows the smallest charge transfer resistance, 17.45 (Ω) compared to other dye cells: 85.38 Ω for blueberry, 68.94 Ω for cranberry, and 33.50 Ω for blackberry respectively. All natural dyes have anthocyanin a pigment responsible for the harvesting of radiant energy. Pomegranate having a large percentage of delphinidin possesses the ability to absorb more light than the other berries under consideration. This greater ability to absorb sunlight results in fast electron (hole) generation and transport, hence a lower hole-electron recombination in the cell. The light absorption is not as good in the berry fruit dye extract resulting in poor electron generation leading to relatively high resistance to the flow of electron at the TiO_2_/dye/electrolyte interphase.

Despite, the observed equivalence between the resistance for all the cell series, they do possess almost similar values. As shown in the I-V curve in the Fig. 8b, the pomegranate dye cell showed the highest efficiency because the low charge transfer resistance acted as the dominant factor affecting the performance of the natural dye sensitized solar cells. Figure 8d shows the Bode phase plot for the NDSSC. The blackberry dye showed a big phase shift in the high frequency region (the maximum frequency: 506 Hz) due to relatively small capacitance (52.8 μF) compared to that of the other dyes. Overall the performance of the pomegranate DSSC is better than blackberry DSSC due to the electron transfer resistance of blackberry dye which is far higher than that of Pomegranate dye.

### Working principle of Dye Sensitized Cell (DSSC)

**Figure 9:**
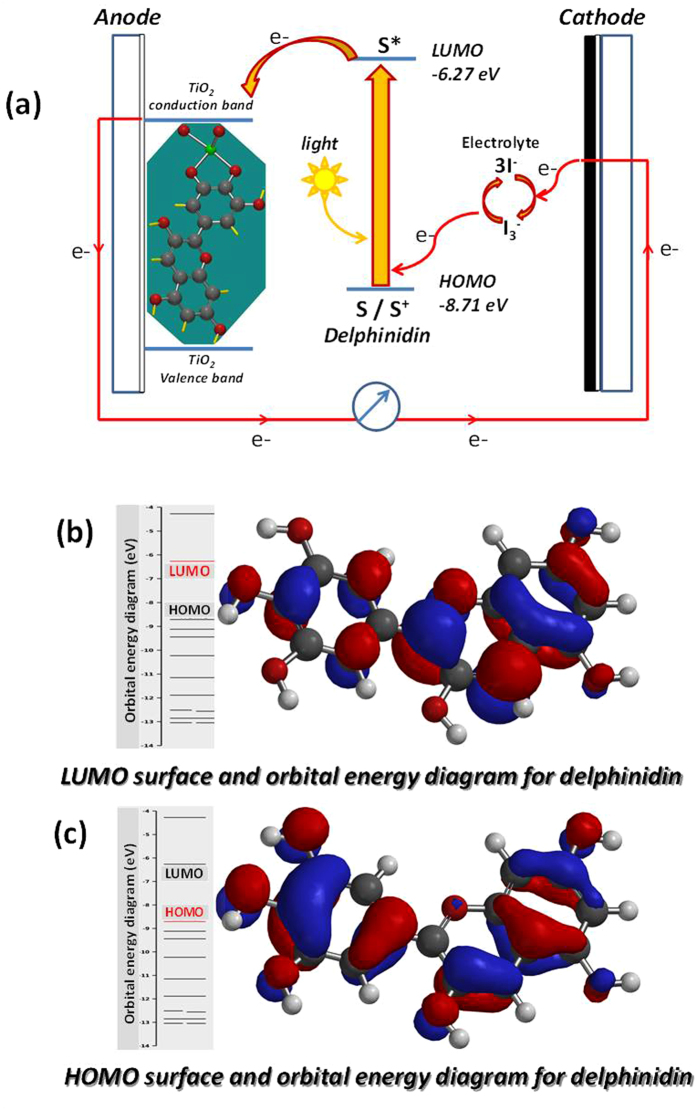
(**a**) Working principles of DSSC with delphinidin; (**b**) HOMO and (**c**) LUMO surface and orbital energy diagram for delphinidin.

Upon illumination, dye molecules (S) adsorbed on the TiO_2_ film absorb photon and are excited from the highest occupied molecular orbitals (HOMO) to the lowest unoccupied molecular orbital (LUMO) state as shown in Fig. 9(a). The photo-excited dye species (S*) injects an electron into the conduction band of TiO_2_ electrode and becomes oxidized (S^+^). The oxidized dye species subsequently accept an electron from the electrolyte (I^−^) and the ground state of the dye (S) is restored. The injected electron percolates through the mesoporous TiO_2_ film to the FTO layer and is transported through an external circuit to a load where the work done is delivered as electrical energy. The electron from the external load diffuses to the cathode where it gets transferred to the electrolyte, (I_3_^−^) so the electrolyte system is regenerated. Density Functional Theory (DFT) calculations were used to optimize the geometry of delphinidin molecule using the software Spartan’ 14 from Wavefunction, Inc. Irvine, CA, USA. These calculations were used to determine the highest occupied molecular orbital (HOMO) and the lowest unoccupied molecular orbital (LUMO) energies of delphinidin dye. The anthocyanidin, delphinidin, was chosen for the calculations because it has been shown that it’s derivative, delphinidin -3, 5-diglucoside is the predominant anthocyanin present in pomegranate dye extract. The calculations gave a result for the HOMO of −8.71 eV and the result for the LUMO of −6.27 eV. The difference in the HOMO and LUMO, which is the energy band gap, was found to be 2.44 eV.

The HOMO and LUMO surfaces and orbital energy diagrams are shown in Fig. 9(b and c) respectively. In Fig. 9(b and c), the blue and red regions represent positive and negative values of the orbitals, respectively.

## Conclusion

Dye extract from pomegranate fruit was utilized as the light-harvesting analog in the fabrication of a low cost, eco-friendly dye-sensitized solar cell. The chemical, structural, morphology and optical properties of the pomegranate/TiO_2_ coated FTO glass were investigated via atomic force microscopy, field emission scanning electron microscopy, energy-dispersive x-ray spectroscopy, uv-vis and fluorescence spectroscopy. The fabricated DSSC showed an enhanced solar-to-electrical energy conversion. Theoretical calculation on delphinidin, an anthocyanin derivative found in pomegranate resulted in a HOMO of −8.71 eV and LUMO of −6.27 which makes it possible for effective electron transfer of charge from the LUMO of the pomegranate into the conduction band of TiO_2_. The regeneration of the dye by the redox electrolyte (I^−^/I_3_^−^) coupling increases the lifetime of the dye. Also the narrower band gap of delphinidin (2.44 eV) increases the intramolecular electronic transition probabilities. The efficiency of the prepared pomegranate sensitized solar cell was *η* = 2.0%, and fill factor FF = 0.41 with the short circuit current (I_*SC*_) and open circuit voltage (V_*OC*_) being 12.2 mA/cm^2^ and 0.39 V respectively. The excellent photovoltaics performance of the solar cell could be attributed to the high concentration of anthocyanins present in pomegranate dye used in the sensitization of the nanocrystalline TiO_2_. The use of graphite as the counter electrode and TiCl_4_ treatment of the photo-anode improved the solar cell efficiency by more than one percent.

## Experimental Methods

### Materials

Titanium dioxide powder (Degussa P-25) was purchased from the institute of chemical education, university of Wisconsin-Madison, department of chemistry, Madison, WI, USA. Fluorine tin oxide (FTO) conducting glass slides were purchased from Harford glass company, Hartford City, Indiana, USA. Sodium Hydroxide (NaOH), acetone (C_3_H_6_O), ethanol (C_2_H_5_OH) and acetic acid (CH_3_CO_2_H) were purchased from Sigma-Aldrich (St. Louis, MS, USA) and were used without further purification.

### Characterization techniques

The morphology of each film was analyzed using field emission scanning electron microscopy (Model FESEM: JSM-7100FA JEOL USA, Inc.). The surface morphological characteristics of the nanocrystalline TiO_2_ were experimentally evaluated using Atomic Force Microscopy (AFM) manufactured by NT-MDT (Model: Solver next, NT-MDT). Absorption spectroscopy was carried out with UV-3600 Plus from Shimadzu, MD, USA. Emission spectroscopy was measured with RF-5301PC from Shimadzu, MD, USA. (Raman Studies was carried A model DXR smart Raman spectrometer (Thermo Fisher Scientific Co., Ltd., USA). ATR spectra were obtained with a Thermo Nicolet iS50 FTIR.) Transmission Electron Microscopy (TEM) images were acquired on JEM-1400 PLUS (JEOL USA, Peabody, Massachusetts, USA). The images were viewed using Digital Micrograph software from GATAN (GATAN Inc., Pleasanton, CA, USA). TiO_2_ paste was printed on FTO glass using WS-650 Series Spin Processor from Laurell Technologies Corporation, PA, USA. Carbon paint used in making cathode slides was purchased from TED PELLA, INC, USA. The cell performance was measured using 150 W fully reflective solar simulator with a standard illumination of air-mass 1.5 global (AM 1.5 G) having an irridance of 100 mW/cm^2^ (Sciencetech Inc.), London, Ontario, Canada. Reference 600 Potentiostat/Galvanostat/ZRA from GAMRY Instruments (Warminster, PA). HOMO and LUMO calculations were carried out using Spartan’14 software from Wavefunction, Inc. Irvine, CA, USA.

### Natural Dye Extraction

A fresh pomegranate and berry fruits were peeled and the juice extracted from the pulp coats using a commercial fruit juice extractor. The extraction was followed by a sequence of filtration, centrifugation, and decantation to remove any precipitate present in the crude extract.

### Fabrication of NDSSC

The electrodes were prepared according to a previously published procedure with some modification^31^. The Supplementary Fig. S1 shows a schematic of the stages involved in the fabrication of the pomegranate dye sensitized solar cell. The working electrode was prepared by depositing a thin film of TiO_2_ on the conductive side of a fluorine doped tin oxide (FTO) glass using a spin coater and annealing the film at 380 °C for 2 hours. The TiO_2_ coated FTO glass was subsequently dipped in TiCl_4_ solution for an hour and annealed again for 30 minutes. The substrate was then immersed in a freshly squeezed pomegranate juice for dye sensitization. To concentrate the sample, pomegranate juice was lyophilized and dissolved in a minimum amount of water before dye sensitization. The counter electrode (cathode) was prepared by either depositing carbon soot on FTO glass slide or painting this glass slide with colloidal graphite. The pomegranate dye sensitized and the carbon electrodes were assembled to form a solar cell by sandwiching a redox (I^−^/I_3_^−^) electrolyte solution as displayed in Supplementary Fig. S1.

## Additional Information

**How to cite this article:** Ghann, W. *et al*. Fabrication, Optimization and Characterization of Natural Dye Sensitized Solar Cell. *Sci. Rep.* **7**, 41470; doi: 10.1038/srep41470 (2017).

**Publisher's note:** Springer Nature remains neutral with regard to jurisdictional claims in published maps and institutional affiliations.

## Supplementary Material

Supplementary Information

## Figures and Tables

**Figure 1:**
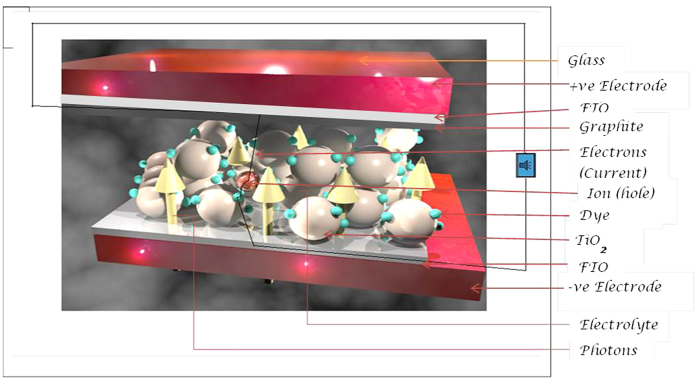
3D-diagram illustrating a magnified cross-section of Dye Sensitized Solar Cell (DSSC).

**Table 1:** Photovoltaic performance for fabricated pomegranate sensitized solar cell in comparison with other fabricated cell from the berry family (Blackberry, Raspberry, Blueberry and Cranberry).

	V_*OC*_ (V)	I_*SC*_ (mA/cm^2^)	V_*mp*_ (V)	I_*mp*_ (mA/cm^2^)	Fill Factor (%)	Efficiency (%)
Pomegranate	0.39	12.20	0.23	8.5	**0**.**41**	**2**.**0**
Blackberry	0.47	11.16	0.20	6.98	**0**.**26**	**1**.**4**
Cranberry	0.41	6.78	0.27	4.31	**0**.**42**	**1**.**2**
Blueberry	0.42	2.72	0.25	1.78	**0**.**38**	**0**.**4**

## References

[b1] Singh G. K. Solar power generation by PV (photovoltaic) technology: A review. Energy 53, 1–13 (2013).

[b2] Chen W. . Efficient and stable large-area perovskite solar cells with inorganic charge extraction layers. Science 350, 944–948 (2015). 26516198 10.1126/science.aad1015

[b3] Yoo K. . Completely Transparent Conducting Oxide-Free and Flexible Dye-Sensitized Solar Cells Fabricated on Plastic Substrates. ACS Nano 9, 3760–3771 (2015). 25769343 10.1021/acsnano.5b01346

[b4] O’Regan B. & Gratzel M. A low-cost, high-efficiency solar cell based on dye-sensitized colloidal TiO_2_ films. Nature 353, 737–740 (1991).

[b5] Narayan M. R. Review: Dye sensitized solar cells based on natural photosensitizers. Renew Sust Energ Rev 16, 208–215 (2012).

[b6] Hao S., Wu J., Huang Y. & Lin J. Natural dyes as photosensitizers for dye-sensitized solar cell. Sol. Energ 80, 209–214 (2006).

[b7] Shalini S., Balasundara prabhu R., Prasanna S., Mallick T. K. & Senthilarasu S. Review on natural dye sensitized solar cells: Operation, materials and methods. Renew Sust Energ Rev 51, 1306–1325 (2015).

[b8] Sugathan V., John E. & Sudhakar K. Recent improvements in dye sensitized solar cells: A review. Renew Sust Energ Rev 52, 54–64 (2015).

[b9] Bahadur K. I., Jyoti N. J., Kumar M. P. & Suman C. Dye-Sensitized Solar cell using extract of Punica Granatum L. Pomegranate (Bedana) as a Natural Sensitizer. Research of J. Chem. Sci. 2, 81–83 (2012).

[b10] Meyer G. J. The 2010 Millennium Technology Grand Prize: Dye-Sensitized Solar Cells. ACS Nano. 4, 4337–4343 (2010). 20731419 10.1021/nn101591h

[b11] Hug H., Bader M., Mair P. & Glatzel T. Biophotovoltaics: Natural pigments in dye-sensitized solar cells Appl. Energy 115, 216–225 (2014).

[b12] Ludin N. A. . Review on the development of natural dye photosensitizer for dye-sensitized solar cells. Renew Sust Energ. Rev. 31, 386–396 (2014).

[b13] Mathew S. . Dye-sensitized solar cells with 13% efficiency achieved through the molecular engineering of porphyrin sensitizers. Nat. Chem. 6, 242–247 (2014). 24557140 10.1038/nchem.1861

[b14] Karki I. B., Nakarmi J. J., Mandal P. K. & Chatterjee S. Absorption Spectra of Natural Dyes and Their Effect on Efficiency of ZnO Based Dye-Sensitized Solar Cells. NJST 13, 179–185 (2013).

[b15] Duffy N. W., Peter L. M., Rajapakse R. M. G. & Wijayantha K. G. U. Investigation of the Kinetics of the Back Reaction of Electrons with Tri-Iodide in Dye-Sensitized Nanocrystalline Photovoltaic Cells. The J. Phys. Chem B. 104, 8916–8919 (2000).

[b16] Hosseinnezhad M., Moradian S. & Gharanjig K. Fruit extract dyes as photosensitizers in solar cells. Curr. Sci. 109, 953–956 (2015).

[b17] Dai Q. & Rabani J. Photosensitization of nanocrystalline TiO_2_ films by anthocyanin dyes. J. of Photochem. Photobiol. 148, 17–24 (2002).

[b18] Dai Q. & Rabani J. Photosensitization of nanocrystalline TiO_2_ films by pomegranate pigments with unusually high efficiency in aqueous medium. Chem Commun. 20, 2142–2143 (2001). 10.1039/b106197f 12240203

[b19] Noda Y., Kaneyuki T., Mori A. & Packer L. Antioxidant activities of pomegranate fruit extract and its anthocyanidins: delphinidin, cyanidin, and pelargonidin. J Agric Food Chem. 50, 166–171 (2002). 11754562 10.1021/jf0108765

[b20] Hou D.-X., Fujii M., Terahara N. & Yoshimoto M. Molecular Mechanisms Behind the Chemopreventive Effects of Anthocyanidins. J Biomed Biotechnol. 2004, 321–325 (2004). 15577196 10.1155/S1110724304403040 PMC1082887

[b21] Cherepy N. J., Smestad G. P., Grätzel M. & Zhang J. Z. Ultrafast Electron Injection: Implications for a Photoelectrochemical Cell Utilizing an Anthocyanin Dye-Sensitized TiO_2_ Nanocrystalline Electrode. J. Phys. Chem B. 101, 9342–9351 (1997).

[b22] Bazargan M. H., Byranvand M., Kharat N. & Fatholahi L. Optoelectron Adv Mat. 5, 360–362 (2011).

[b23] Li N., Pan N., Danhong Li & Lin S. Natural Dye-Sensitized Solar Cells Based on Highly Ordered TiO_2_ Nanotube Arrays. Int. J. Photoenergy. 598753, 1–5 (2013).

[b24] Sivaranjani K. & Gopinath C. S. Porosity driven photocatalytic activity of wormhole mesoporous TiO_2_-xNx in direct sunlight. J. Mater. Chem. 21, 2639–2647, (2011).

[b25] Kumar P. M., Badrinarayanan S. & Sastry M. Nanocrystalline TiO_2_ studied by optical, FTIR and X-ray photoelectron spectroscopy: correlation to presence of surface states. Thin Solid Films 358, 122–130 (2000).

[b26] Zhang J.-Y. . Nanocrystalline TiO_2_ films studied by optical, XRD and FTIR spectroscopy. J. Non-Cryst. S 303, 134–138 (2002).

[b27] Kudin K. N. . Raman Spectra of Graphite Oxide and Functionalized Graphene Sheets. Nano Let. 8, 36–41 (2008). 18154315 10.1021/nl071822y

[b28] Maiaugree W. . A dye sensitized solar cell using natural counter electrode and natural dye derived from mangosteen peel waste. Sci Rep. 5, 15230, (2015). 26458745 10.1038/srep15230 PMC4602286

[b29] Voïtchovsky K. . *In Situ* Mapping of the Molecular Arrangement of Amphiphilic Dye Molecules at the TiO_2_ Surface of Dye-Sensitized Solar Cells. ACS Appl. Mater. Interfaces 7, 10834–10842 (2015). 25936429 10.1021/acsami.5b01638

[b30] Lim S. P., Pandikumar A., Lim H. N., Ramaraj R. & Huang N. M. Boosting Photovoltaic Performance of Dye-Sensitized Solar Cells Using Silver Nanoparticle-Decorated N, S-Co-Doped-TiO_2_ Photoanode. Sci Rep. 5, 11922 (2015). 26146362 10.1038/srep11922 PMC4491728

[b31] Amadi L. . Creation of Natural Dye Sensitized Solar Cell by Using Nanostructured Titanium Oxide. Nanosci. Nanoeng. 3, 25–35 (2015).

