# Supplementary material for: Fabrication, Optimization and Characterization of Natural Dye Sensitized Solar Cell
Source: Sci Rep. 2017 Jan 27;7:41470. doi: 10.1038/srep41470 (PMC5270247; doi:10.1038/srep41470)
Supplement: Supplementary Information [file srep41470-s1.pdf]

Supporting Information

Fabrication, Optimization and Characterization of Natural Dye Sensitized Solar Cell

William Ghann, Hyeonggon Kang,Tajbik Sheikh, Sunil Yadav, Tulio Chavez-Gil, Fred Nesbitt and Jamal Uddin*

Coppin State University, Center for Nanotechnology, Department of Natural Sciences, 2500 W. North Ave., Baltimore, MD

*juddin@coppin.edu

Solar Cell Fabrication

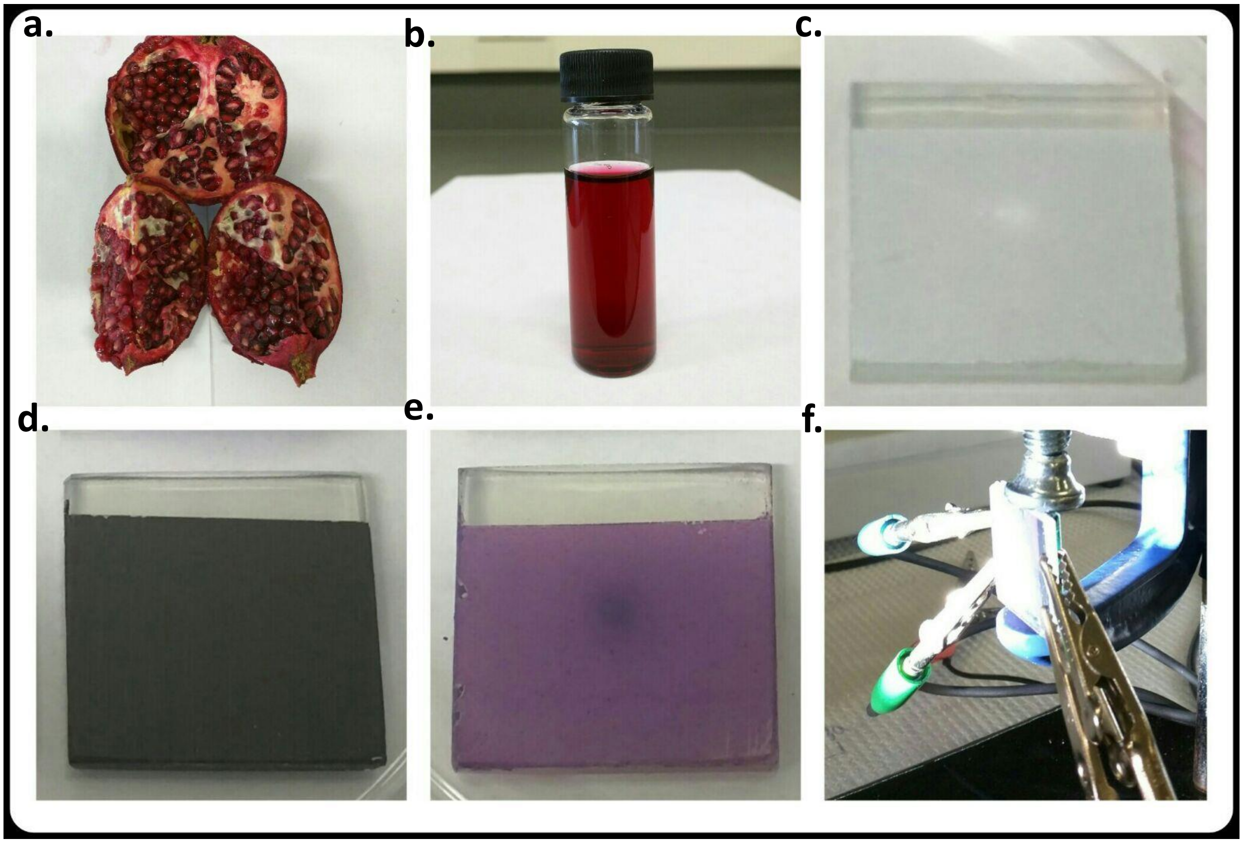

Figure S1: Cell fabrication: (a) sliced pomegranate fruit; (b) freshly prepared pomegranate dye; (c) Bare TiO_2 coated FTO glass; (d) carbon coated counter electrode; (e) pomegranate dye sensitized TiO_2 slide; (f) dye sensitized solar cell under illumination.

Emission Studies on Pomegranate dye

Emission studies were carried out on the pomegranate dye and the results are displayed in Supplementary Fig. S2. The results are presented in 2-D (a&b) and 3D (c) format. Two major peaks, one at 450 nm and the other at 610 nm are observed. The result of the measurement is a validation that pomegranate dye absorbs and emits photon over a wide range of wavelengths. This higher light harvesting abilities results in greater charge generation.

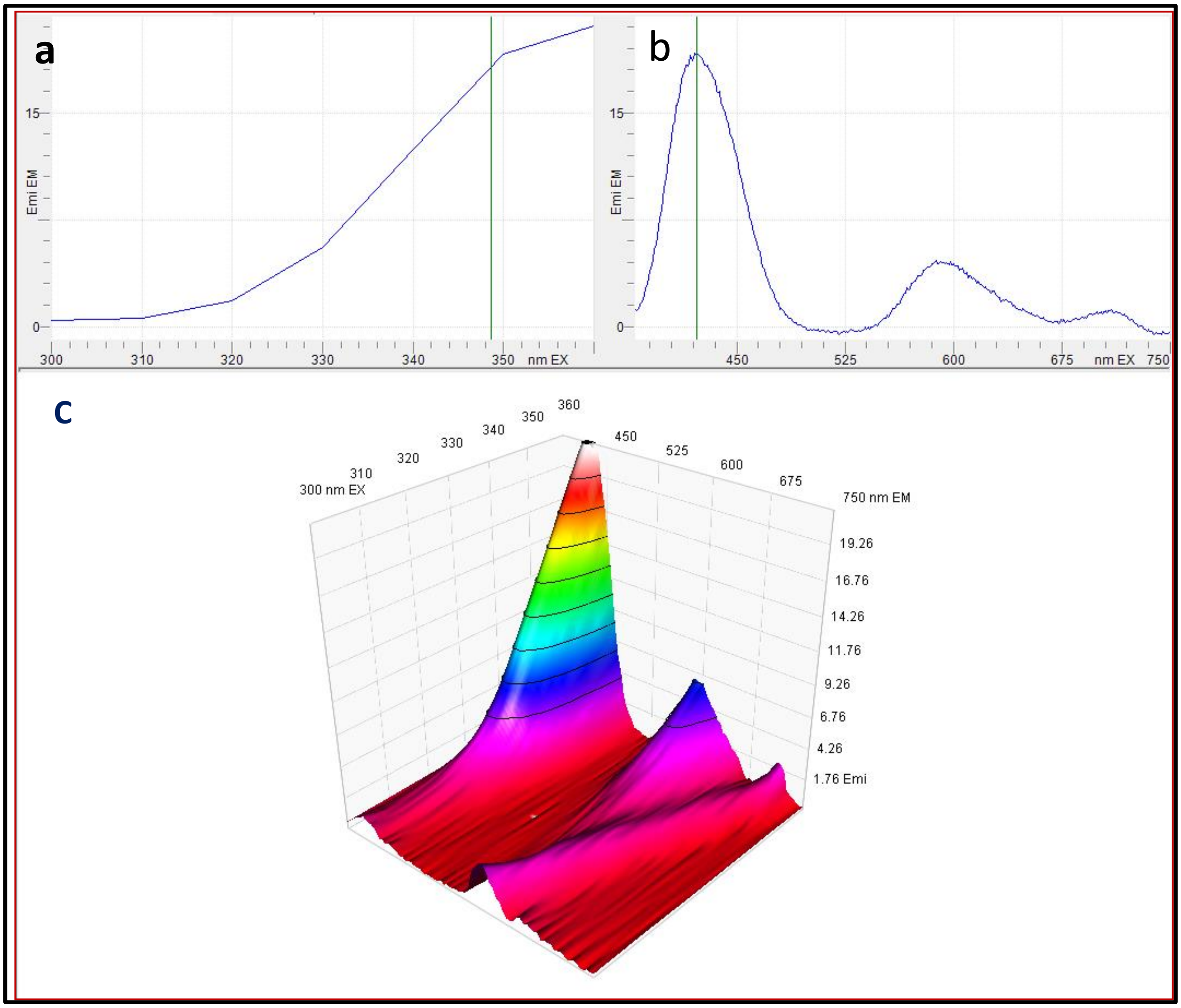

Figure S2: Emission spectra of pomegranate dye extract: (a) 2D excitation display; (b) 2D emission display (top); (c) 3D emission spectrum (bottom)

Atomic Force Microscopy (AFM) Images

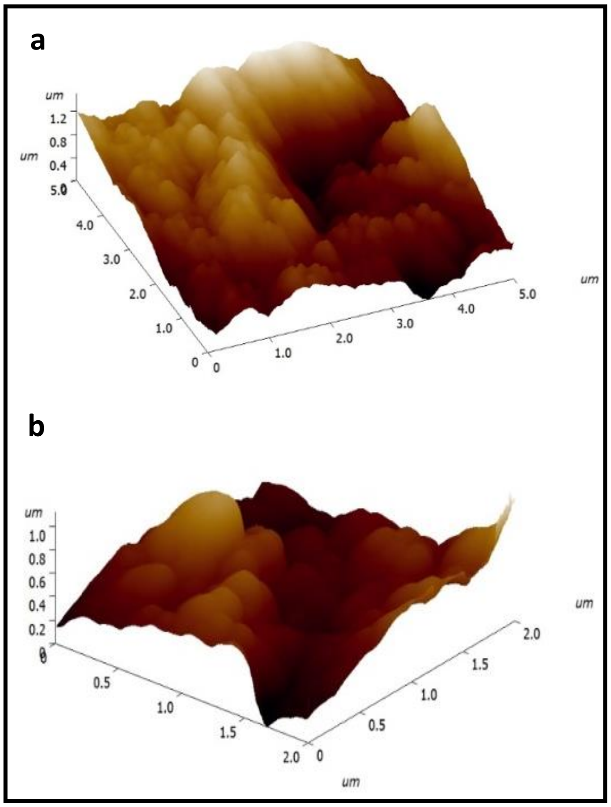

Figure S3: Three dimensional AFM images of bare TiO_2 and pomegranate sensitized TiO_2 on FTO glass: (a) bare TiO_2 sample (5 µm x 5 µm); (b) pomegranate Dye/TiO_2 sample (2 µm x 2 µm)

Field Emission Scanning Electron Microscopy (FESEM) Image Analysis

Supplementary Figure S4 (a) is a cross-sectional image of the Dye/TiO_2/FTO glass sample. To carry out this imaging study, a slice of Dye/TiO_2/FTO glass sample was affixed on the FESEM stage holder using a piece carbon tape and carbon paste. The cross-sectional image was taken with the FESEM (model, JSM-7100F, Jeol.com) and layers of the image analyzed by Energy Dispersive X-ray Spectroscopy (EDS). The results of the EDS analysis are displayed in the Supplementary Fig. S6 (c). Map analysis of pomegranate sensitized FTO slide is depicted in Supplementary Fig. S4 (b). The elements observed in the sample were carbon and Titanium. Carbon is a constituent of the organic dye and Titanium, a structural element of the semiconductor. Cracks in the titanium dioxide layer are clearly defined.

Supplementary Figure S4 (c) is a display of the map analysis of bare FTO glass slide which shows Tin to be the predominant element present. Tin is a major component of the Fluorine doped tin oxide (FTO) conductive substrate. The granular nature of the FTO is also clearly evident in the FESEM image of the bare FTO glass slide. Such minute details and grains are not observed with naked eye.

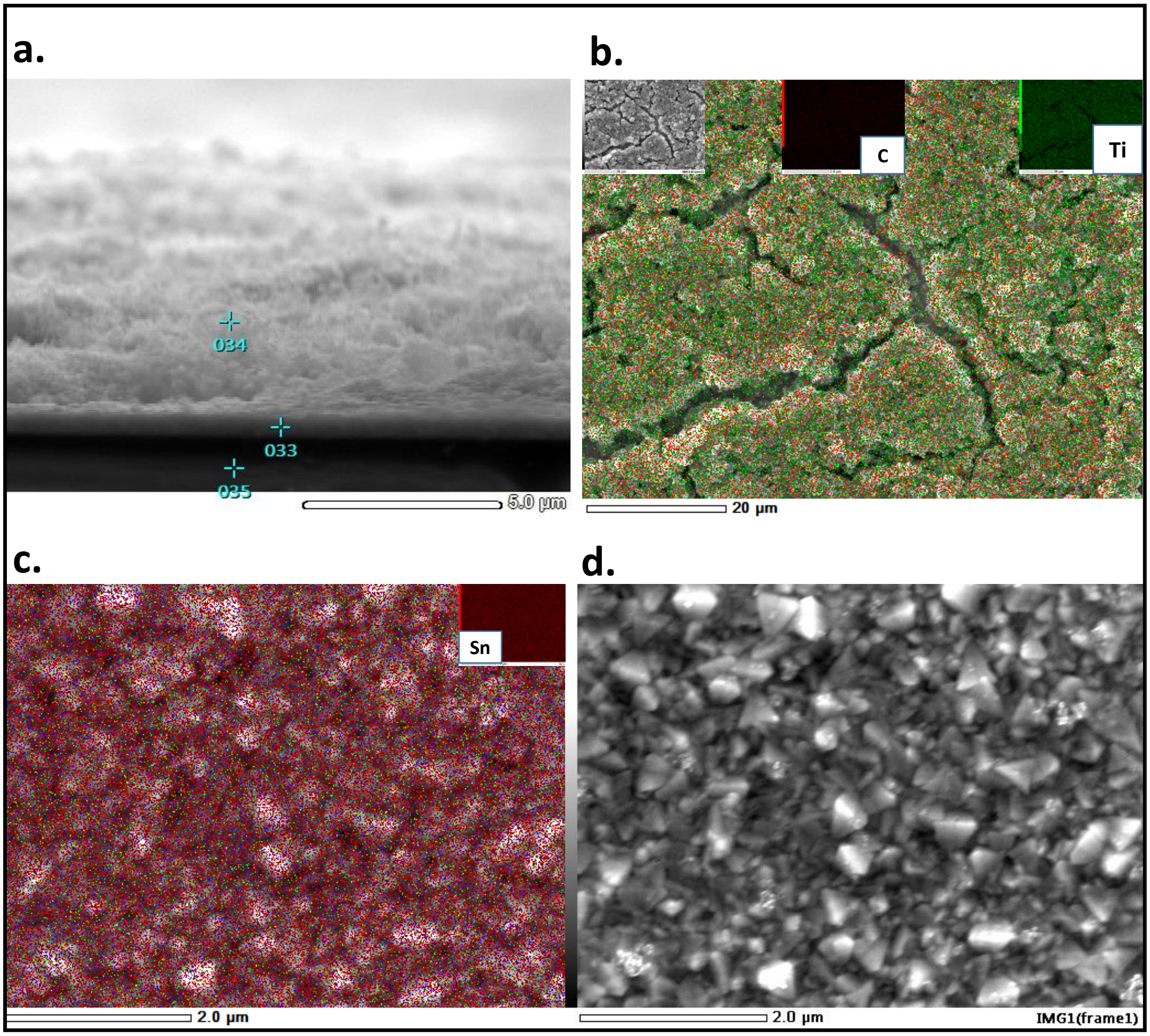

Figure S4: Cross sectional imaging and map analysis of samples: (a) cross sectional image of pomegranate dye sensitized TiO_2 film on FTO glass; (b) map analysis of pomegranate dye sensitized TiO_2 film on FTO glass;(c) map analysis of FTO Glass; (d) FESEM image of FTO Glass

Supplementary Figure S5 (a) shows an image of FTO slide partially covered with TiO_2 film. The nanocrystalline nature of the TiO_2 is clearly revealed in the image. The large surface area necessary for supporting the dye molecules is attained by way of the nanocrystalline nature of TiO_2. Supplementary Figure S5 (b) shows the nanocrystalline mesoporous TiO_2 film with a layer of pomegranate dye. A line profile (Supplementary Fig. S5 (d)) of a partially sensitized TiO_2 film (Supplementary Fig. S5 (c)) reveals the relative intensities of the Titanium and Carbon present in the sample. This analysis was carried out to distinguish the dye sensitized TiO_2 from the bare TiO_2 at the elemental level. The intensity of carbon was greater than that of Titanium in the portion of the film covered with dye.

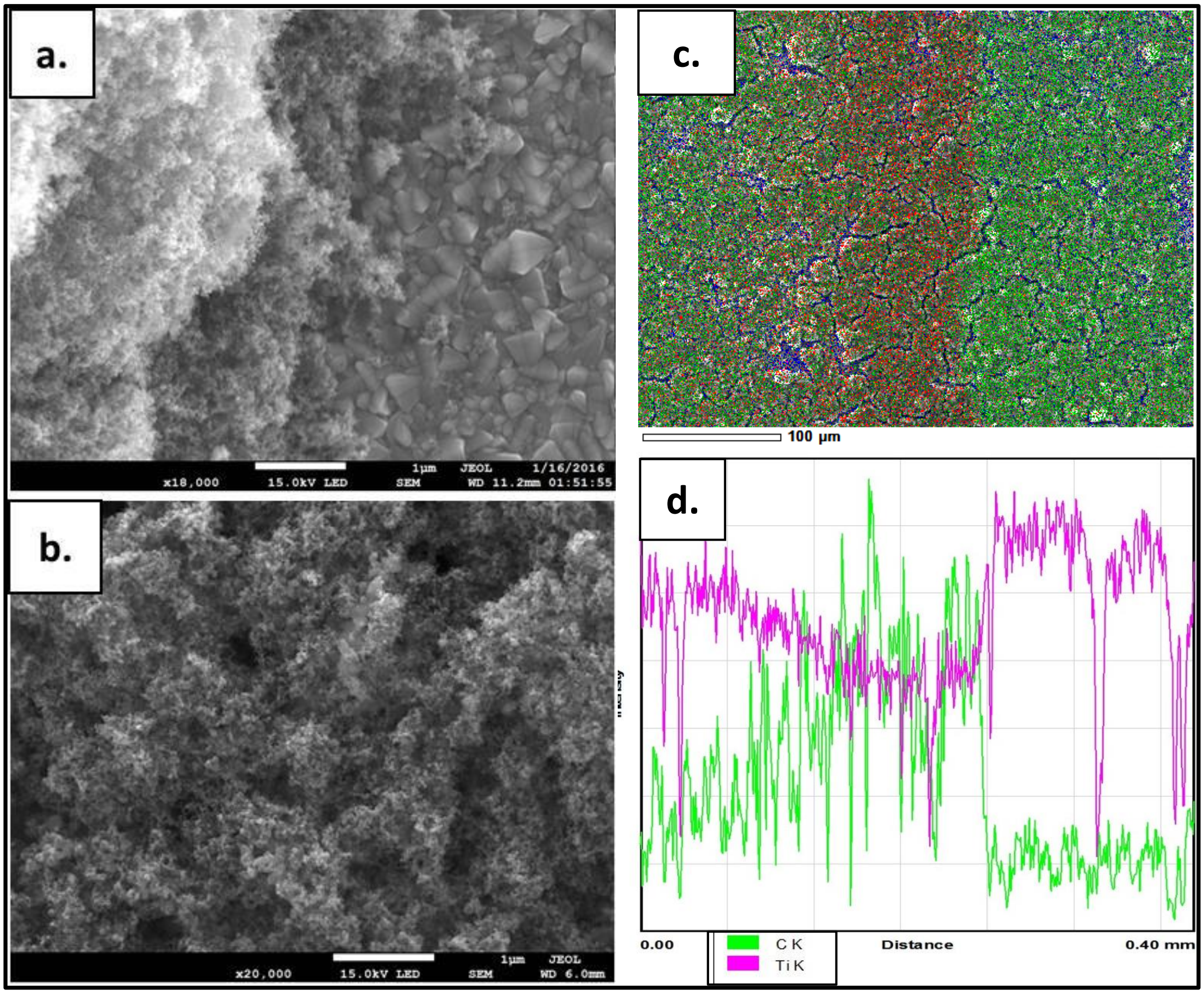

Figure S5: SEM images of titanium dioxide coated FTO slides and line profile of TiO_2 coated FTO slide with a partial layer of pomegranate dye:(a) TiO_2 on FTO slide; pomegranate sensitized FTO slide;(c) TiO_2 coated FTO slide with a layer of Pomegranate;(d) Line profile of TiO_2 coated FTO slide with a partial layer of pomegranate dye.

Energy dispersive X-ray Spectroscopic Analysis

Supplementary Figure S6 shows the EDS measurements of bare glass without FTO (a), FTO coated glass (b) and dye sensitized TiO_2 film on FTO glass (c). In In the absence of pomegranate dye (Supplementary Fig. S6 (a &b)), the carbon peak is negligible. Supplementary Figure S6 (c) exhibit a spectrum with huge peaks for Carbon and Titanium which are as a result of pomegranate dye and titanium dioxide, respectively. The result displayed in Supplementary Fig. S6 (a&b) therefore confirms that the carbon peak detected in Supplementary Fig. S6 (c) is as a result of the organic dye.

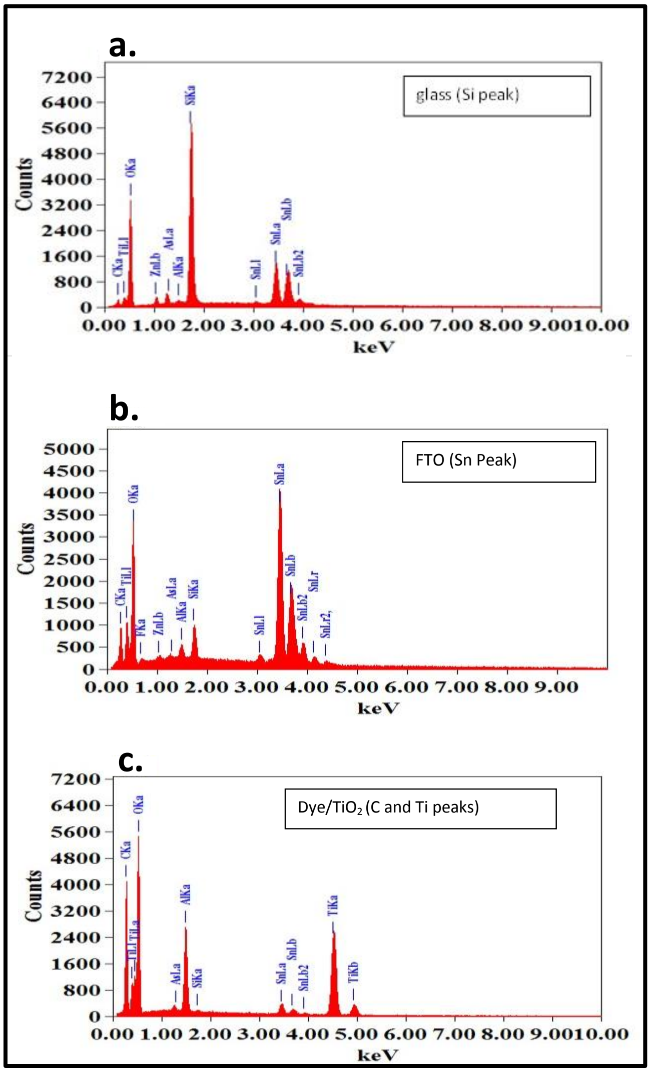

Figure S6: Energy dispersive X-ray spectroscopic (EDS) studies: (a) glass without FTO; (b) FTO coated glass; (c) pomegranate dye sensitized TiO_2 film on FTO glass

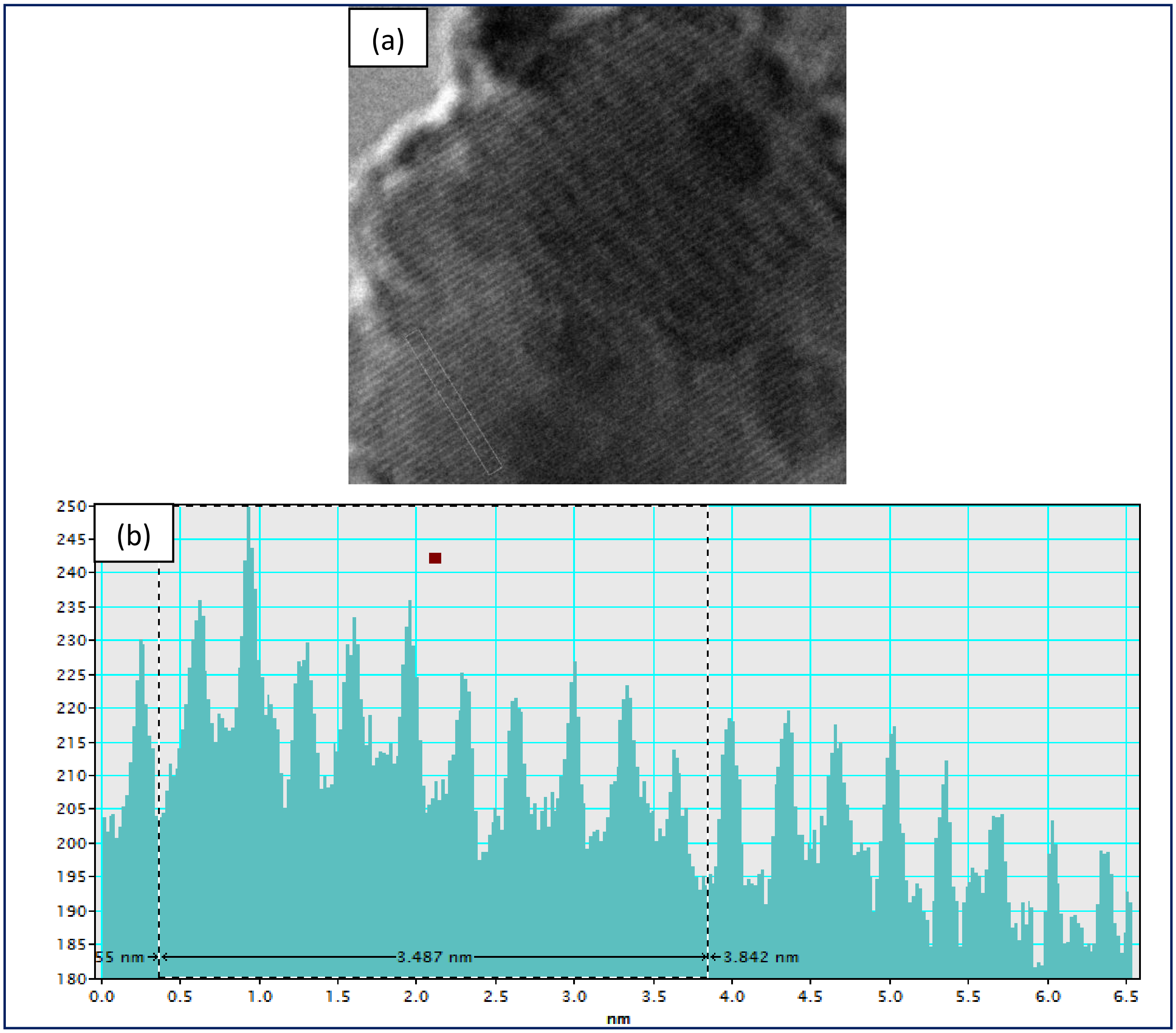

Figure S7: Cross section analysis for the calculation of the d-spacing value from the white box area of pomegranate dye/TiO_2 TEM image: (a) TEM image (b) cross section analysis.

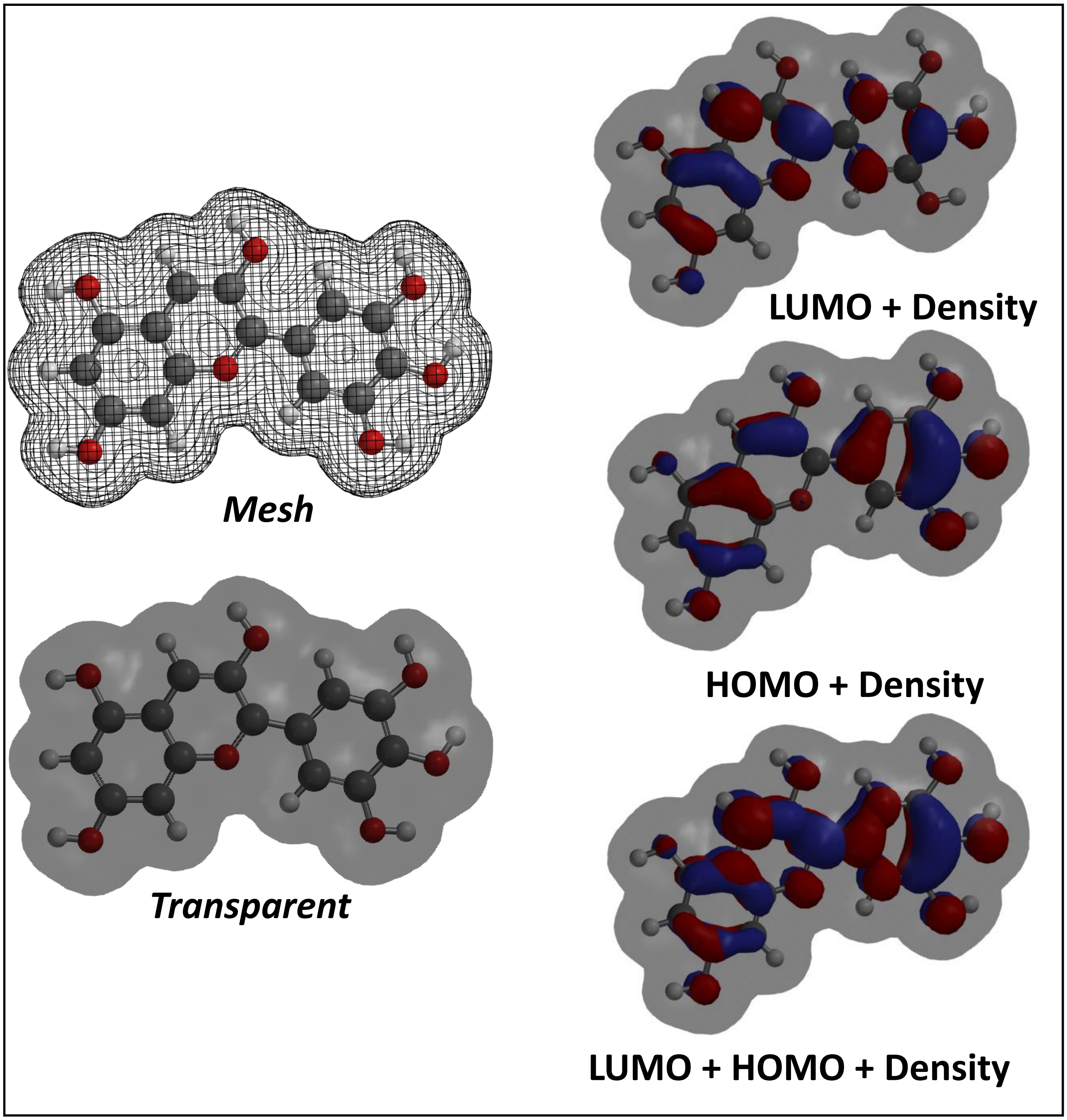

Figure S8: The HOMO and LUMO distribution pattern of delphinidin, a derivative of anthocyanin
